# Role of Stem Cells and Stem Cell Markers in Oral Potentially Malignant Disorders and Malignant Transformation: A Systematic Review

**DOI:** 10.1155/sci/9371262

**Published:** 2026-03-02

**Authors:** Nadisha S. Piyarathne, Gayani S. Nawarathna, W. J. Wijesingha, Udari Abeyasinghe, P. V. Kalani Hettiarachchi

**Affiliations:** ^1^ Institute of Dentistry, School of Medicine, Medical Science and Nutrition, University of Aberdeen, Aberdeen, AB25 2ZR, UK, abdn.ac.uk; ^2^ Center for Research in Oral Cancer, Faculty of Dental Sciences, University of Peradeniya, Kandy, 20400, Sri Lanka, pdn.ac.lk; ^3^ Department of Basic Sciences, Faculty of Dental Sciences, University of Peradeniya, Kandy, 20400, Sri Lanka, pdn.ac.lk; ^4^ Postgraduate Institute of Medicine, University of Colombo, Colombo, 00700, Sri Lanka, cmb.ac.lk; ^5^ Department of Oral Pathology, Faculty of Dental Sciences, University of Peradeniya, Kandy, 20400, Sri Lanka, pdn.ac.lk; ^6^ Frazer Institute, Faculty of Health, Medicine and Behavioural Sciences, The University of Queensland, Woolloongabba, 4102, Queensland, Australia, uq.edu.au

**Keywords:** malignant transformation, oral potentially malignant disorders, stem cell marker, stem cells

## Abstract

Oral potentially malignant disorders (OPMDs) have varying risk of malignant transformation (MT), yet the underlying mechanisms remain unclear. Recent evidence suggest emerging role of stem cells in carcinogenesis. This systematic review aimed to synthesizes current knowledge on the role of stem cells in OPMD and MT. Review protocol was developed in accordance with PRISMA 2020 guidelines and registered with PROSPERO. Literature searches identified 4882 records from PubMed, Scopus, Embase, and Web of Science databases; from these, *n* = 97 primary research studies were selected via two stage screening. Data extraction and narrative synthesis was conducted according to synthesis without meta‐analysis (SWiM) guidelines. Methodological quality was assessed using Joanna Briggs Institute (JBI) critical appraisal checklists. Studies included in this review were published between 2006–2025, where majority of the research were from India and China. Immunohistochemistry (IHC) was used to identify stem cell biomarkers in tissue samples, most studies demonstrated that higher expression of stem cell markers (CD44, ALDH1, HELLS, TARIF, SOX2, NANOG, and CD147) correlated with severity of epithelial dysplasia. Longitudinal data identified ALDH1 and Bmi‐1 as promising prognostic biomarkers linked to MT. Evidence from cell culture and animal model experiments suggested potential therapeutic applications of stem cells and their exosomes in haltering the progression of OPMD. Notably, a clinical trial incorporated stem cell markers as surrogate end points for evaluating treatment options. While findings underscore the prognostic and therapeutic relevance of stem cells in OPMD, lack of prospective designs in biomarker validation and absence of clinical trial evidence on stem cell therapies limit clinical applicability.

## 1. Introduction

Oral potentially malignant disorders (OPMDs) represents a heterogeneous group of clinical conditions characterized by an increased risk of malignant transformation (MT) into oral squamous cell carcinoma (OSCC). The worldwide prevalence of OPMD was estimated at 4.47% [1], with regional variations as high as threefold such as in South India with a rate of 13.28% [2]. OPMD include several clinical subtypes, common presentations being oral lichen planus (OLP), leukoplakia (OL), erythroplakia (OE), and oral submucous fibrosis (OSMF). Oral epithelial dysplasia (OED) is the histological entity preceding invasive OSCC and may be present with or without visible clinical features [3]. OSCC remains the most prevalent histological subtype of the malignancies arising in the oral cavity [4].

A meta‐analysis estimated that the overall MT rate of OPMD was 7.9% [5]. A nationwide analysis from Taiwan reported MT rates specific for clinical subtypes, the highest MT rate was for verrucous hyperplasia (24.5 per 1000 person years) followed by OSMF, erythroplakia, OLP, and leukoplakia [6]. Despite extensive research for decades, the exact mechanistic derivers of MT of OPMD remain ambiguous. Studies have demonstrated that the severity of epithelial dysplasia [7], size and site of lesions, female gender, nonhomogeneous appearance, and behavioral factors such as continuation of risk habits as risk factors for the MT of OPMD [8]. As oral carcinogenesis is a multistep process, progressive accumulation of genetic and environmental stresses could drive the MT. However, the variability observed in MT rates in different clinical entities of OPMDs iterates the fact that underexplored factors may contribute to the development of OSCC in OPMD.

Recent research has identified the emerging role of stem cells in carcinogenesis. The term cancer stem cells (CSCs) was coined to describe a sub population of cells with the potential for self‐renewal, differentiation, therapy resistance, and development of multiple and recurrent forms of cancer [9]. Role of stem cells have been implicated in key cancer hallmarks, including immune evasion, metastasis, recurrence, and resistance to conventional therapies [10]. Their phenotypic plasticity, modulated by the tumor microenvironment, further amplifies the clinical significance.

Despite the hypothesis that CSCs are responsible for de novo appearance of cancers, their role in the MT of OPMD remains underexplored. Some studies have proposed that a distinct CSC subset with specific genetic alterations may be the driving force behind the MT in OPMDs [11, 12]. Further, evidence on the different applications of stem cell markers in the diagnosis and risk assessment of OPMD, assessing the stemness properties of nonmalignant cells, and therapeutic applications remains vague. To address this gap, the present systematic review aims to identify the published literature and critically appraise the evidence on the role of stem cells and stem cell markers in the context of OPMD and their MT.

## 2. Methodology

### 2.1. Protocol and Registration

This systematic review adhered to the Preferred Reporting Items for Systematic Reviews and Meta‐Analyses (PRISMA‐2020) guidelines [13, 14] and was registered with PROSPERO (CRD42024577426). The research question was defined according to the PICOS format: population (OPMD), intervention (stem cells or stem cell markers), comparator (healthy controls or OSCC), outcome (MT), and study design primary research (including observational, interventional, or experimental designs).

### 2.2. Data Sources

A comprehensive systematic electronic literature search was conducted in September 2024 across Pubmed, Web of Science, Embase, and Scopus databases. The reference lists of selected records were screened for further studies. The literature search was updated in October 2025, covering the same databases with limitations applied from 2024 onwards.

### 2.3. Literature Search

Keywords related to stem cells and OPMD were combined with AND/OR Boolean to generate the search syntax. The search was conducted by two reviewers independently (Nadisha S. Piyarathne and Gayani Nawarathne). Search strategy used for the Pubmed database was (“stem cell ^∗^”[Title/Abstract] OR “stem cell marker ^∗^”[Title/Abstract] OR “cancer stem cells”[Title/Abstract] OR “mesenchymal stem cells”[Title/Abstract] OR “pluripotent stem cells”[Title/Abstract] OR “malignant stem cells”[Title/Abstract] OR “carcinoma stem cells”[Title/Abstract]) AND (“oral potentially malignant disorders”[Title/Abstract] OR “OPMD”[Title/Abstract] OR “oral cancer”[Title/Abstract] OR “oral squamous cell carcinoma”[Title/Abstract] OR “oral submucous fibrosis”[Title/Abstract] OR “oral lichen planus”[Title/Abstract] OR “Leukoplakia”[Title/Abstract] OR “oral dysplasia”[Title/Abstract] OR “oral pre cancer”[Title/Abstract]). Keywords related to individual stem cell markers (CD44, ALDH1, HELLS, TARIF, SOX2, NANOG, and CD147) were incorporated in the updated literature search. Similar search syntaxes were used for the Scopus, Embase, and Web of Science databases.

### 2.4. Screening and Study Selection

**Figure 1:**
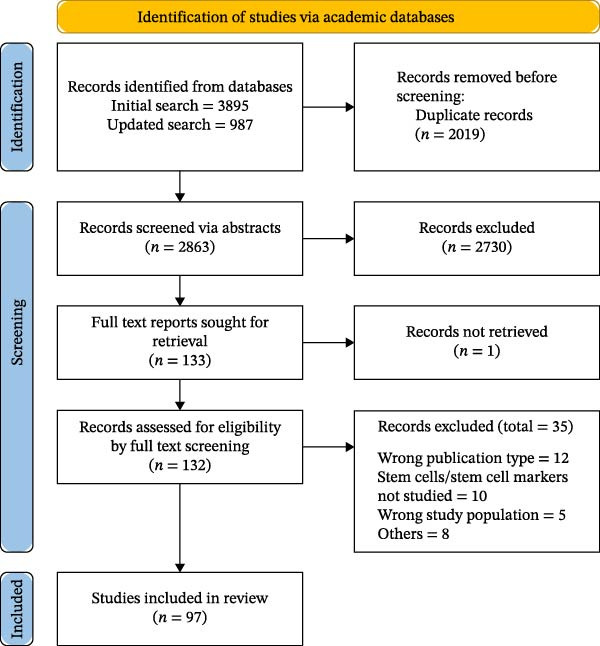
PRISMA flow chart depicting screening and study selection (initial literature search was conducted in September 2024, updated literature search was conducted in October 2025).

Retrieved abstracts were exported and managed using Rayyan.ai and Zotero software. Following de‐duplication, all records were screened by two independent reviewers (Nadisha S. Piyarathne, Gayani Nawarathne, and W. J. Wijesingha). Following title and abstract screening, records were selected for the full‐text screening stage. Any disagreements during the screening and study selection process were resolved through discussion and involvement of a third reviewer (Kalani Hettiarachchi). Screening and study selection is summarized using PRISMA 2020 flow chart (Figure 1).

Screening and study selection at all stages was conducted using predefined selection criteria. The inclusion criteria were (1) original research published in English, (2) studies including any biological samples, and (3) any type of observational or interventional study design, including studies done on OPMD/ OSCC cell lines, samples from OPMD patients and animal models of OPMD/OSCC. Exclusion criteria were (1) secondary studies such as all review types, meta‐analysis, case series, case report, conference proceedings, abstracts, (2) studies that have not reported data on stem cells or stem cell markers and (3) studies not including patinets with OPMD or OED.

### 2.5. Reviewer Calibration

Four reviewers (Nadisha S. Piyarathne, Gayani Nawarathne, W. J. Wijesingha, and Kalani Hettiarachchi) extracted and analyzed data from five randomly selected papers for training and calibration. Once calibration was achieved, reviewers extracted data from each paper independently and blinded to one another’s scores. Disagreements were resolved through discussion and when necessary, with the involvement of a third reviewer (Nadisha S. Piyarathne and Kalani Hettiarachchi).

### 2.6. Data Extraction

Variables extracted from each included article were: first author, published year, title, type of sample, study design, sample size, stem cell experiment, stem cell biomarkers, results and conclusions relevant to stem cells, and stem cell biomarkers with their association to MT. The data were recorded and summarized using a customized Microsoft Excel spreadsheet.

### 2.7. Data Analysis and Synthesis of Evidence

Data synthesis was conducted according to SWiM guidelines [15]. Studies were organized into groups according to evidence type (observational studies with a single time point analysis, observational studies with longitudinal analysis, cell culture, and animal model experiments). When the studies have used combined methodologies, evidence for each section was extracted and reported separately. Data analysis was conducted using Microsoft Excel software.

### 2.8. Quality Assessment

Risk of bias assessment of all included studies was conducted using Joanna Briggs Institute (JBI) Critical Appraisal checklists for each study design. Quality assessment was conducted by at least two reviewers blinded to each other’s scores (Nadisha S. Piyarathne, Gayani Nawarathne, and Kalani Hettiarachchi). Disagreements were resolved through discussion and via a third reviewer (Nadisha S. Piyarathne and W. J. Wijesingha). Since different study designs were included, critical appraisal tools for case control, cohort, randomized control trials, and analytical cross‐sectional studies were employed. Experimental study designs were assessed using the checklist for analytical cross‐sectional studies. Letter “Y” was given to criteria that were satisfactory, “N” was given to criteria that was not satisfactory, and “U” was given to criteria that were unclear. For each checklist, predetermined cut‐off values were used to grade the studies as good (G), fair (F), and poor (P). Cut‐off values for grading criteria for each checklist are given under table footnotes in the results section.

## 3. Results

### 3.1. Characteristics of the Included Studies

The initial literature search identified a total of 3895, whereas 987 records were retrieved from the updated literature search. From the initial search, *n* = 80 studies were selected to be included. From the updated literature search conducted in October 2025, *n* = 17 primary research studies were selected to be included, details of screening and study selection are presented in Figure 1.

The included studies were published between 2006 and2025, with India 28.9% (*n* = 28/97) and China 29.9% (*n* = 29/97) contributing to most of the research. Out of the total, 56.7% (*n* = 55/97) used analytical cross sectional study design, 30.9% (*n* = 30/97) were case control design, while 11.3% (*n* = 11/97) used cohort designs with follow up. There was one randomized controlled trial, and four mixed method studies. Regarding the risk of bias assessment, 72.2% % (70/97) were rated as fair (F), and 20.6% (20/97) and 7.2% (7/97) were rated as good (G) and poor (P), respectively.

### 3.2. Evidence From Observational Studies Using Clinical Samples From Patients

More than half of the included studies used observational study designs, reporting data on stem cell biomarkers in clinical samples. Studies have used immunohistochemistry (IHC) as the primary technique to visualize tissue biomarkers. Findings of the observational studies with a single time point analysis are summarized in Table 1.

**Table 1:** Observational studies reporting stem cell biomarkers in clinical samples.

Study reference	Study groups	Sample size	Stem cell markers	Main findings	QA
[16]	HNSCC, lymph node metastasis, OED, NOM	47	HELLS	Low level of HELLS expression was detected in the basal cell layer of the normal oral mucosa, moderate level was seen in dysplasia and high levels in both HNSCC and lymph node metastasis tissue samples	F

[17]	Leukoplakia, normal	112	CD44	A statistically significant overexpression of membranous CD44 was observed compared to healthy mucosa	F

[18]	OSCC, OED	60	CD44 CD24	66.7% of dysplasia and 65%–80% of OSCC cases showed high expression of CD24 and CD44. Significant correlation between CD24 and CD44	F

[19]	NOM, oral hyperkeratosis (OHK), OLP, OED	70	Integrin β1, NG2, Notch 1, Keratin 15	Markers did not identify individual stem cells, but K15 expression may have diagnostic value for OED	F

[20]	OSCC, OED, NOM	78	TRAF1, Bmi‐1, ALDH1, Lin28B	TRAF1 is highly expressed in OSCC tissues and associated with cancer stem cell markers of ALDH1, Lin28, and BMI‐1. TRAF1 as a NF‐κB upstream regulator may be involved in inflammation‐induced cancer stem cell characteristics	F

[21]	OSCC, oral leukoplakia (OL), NOM	56	ALDH 1/2	Significant higher expression in endothelium (blood vessels) of OSCCs compared to the OLs and not expressed in NOM. No significant difference between OL and NOM	F

[22]	OSCC, OED, NOM	98	NICD, Hes‐1, c‐Myc	Significant increase in the markers with disease severity. No significant correlation between the markers. Double positivity of NICD/c‐Myc and Hes‐1/c‐Myc correlated with worse prognosis	F

[23]	OSCC, oral leukoplakia (OLK), NOM	80	PD‐1, PD‐L1, TIM‐3, CTLA‐4, CD8, IFN‐γ, IL‐2, TGF‐β, EOMES, T‐bet	Significant difference and increased expression of CD8, CD86, and PD‐1 in OSCC and OLK, these markers correlated with clinical stage of OSCC. In OLK, the expression of PD‐L1 and CD86 was higher among patients with severe epithelial dysplasia when compared with patients without epithelial dysplasia. And the expression of PD‐L1 and CD86 of OLK tissues coming from lingual was higher than from gingival origin	F

[24]	Oral erythroplakia Group 1‐untransformed Group 2‐single MT Group 3‐multiple MT	34	ALDH1, Bmi‐1	ALDH1 expression significantly increased from group 1 to group 3. Significant differences in both and either of ALDH1 and Bmi1 positivity between the groups was noted	F

[25]	OSMF NOM	91	Ki67, SOX2, Bmi1	Increased expression of Ki67 in the OSMF compared to NOM. Ki 67 expression correlated with severity of OSMF. SOX2 and Bmi1 are positively correlated with Ki67. Expression of SOX2 and Bmi1 was higher in OSMF with dysplasia than in OSMF with epithelial hyperkeratosis	F

[26]	OSCC, oral leukoplakia	51	ALDH 1	Significantly increased expression was observed with disease severity	F

[27]	OSCC, oral leukoplakia, NOM	82	Nestin	Nestin expression was observed in both normal and OSCC samples with six‐fold higher expression in OSCC compared to NOM. The cytoplasmic and membranous expression gradually decreasing from leukoplakia without dysplasia to leukoplakia with mild/moderate dysplasia, and further decreased in leukoplakia with severe dysplasia	F

[28]	OSCC, oral leukoplakia (OL), NOM	56	SOX2 and OCT3/4	All samples were positive for gene expression of both markers. SOX2 expression was statistically higher in OSCC than OL and NOM, where in the OL group increased expression with dysplasia grading. OCT3/4 was only expressed in OSCC	G

[29]	OSCC, OSMF, OSMF‐MT, NOM	100	E‐cadherin, N‐cadherin, pan‐ cytokeratin (PanCK), vimentin, α‐SMA, CD44	Significantly decreased expression of E‐cadherin and PanCK, while a significant increase in the expression of N‐cadherin, vimentin, α‐SMA, and CD44 markers were observed in OSMF‐MT and OSCC groups compared to OSMF, in both at protein and gene expression levels. CD44 expression was noticeably higher in OSMF‐MT group than in OSCC	F

[30]	OSCC, OED	60	OCT4, SOX2	Significant increased expression of both markers were observed in OSCC compared to OED	F

[31]	OSCC, OED, NOM	84	NANOG	Significantly higher expression was observed in OSCC compared to OED. Though the mean score was higher in high‐risk OED than in low‐risk OED, a statistical significance could not be obtained	F

[32]	OSCC, OED	55	NANOG	Increased expression was observed with the grade of dysplasia. Positive NANOG expression was significantly associated with tobacco and alcohol consumption, and was more frequent in pN0 tumors, and early clinical stages	G

[33]	OSCC, oral leukoplakia (OL)	50	CD147	CD147 was upregulated significantly in the moderately and severely dysplastic OLs than in the mildly dysplastic and nondysplastic Ols. Also, CD147 was upregulated significantly in the mildly dysplastic and non‐dysplastic OLs than in the normal oral epithelium	G

[34]	OLP, oral lichenoid lesions, oral leukoplakia and chronic inflammation	64	ALDH1	ALDH1 expression in epithelium was low in all groups without difference among them. ALDH1^+^ cells in the lamina propria were higher for lichen planus, followed by leukoplakia, and lichenoid lesions.	G

[35]	OSCC, OED	140	SALL4	SALL4 positivity was observed to be significantly higher in the tumor cells of OSCC. However, in leukoplakia with dysplasia the SALL4 expression was weak	F

[36]	OSCC, OED	95	P63	P63 positivity was found in 100% cases of dysplasia and 96.3% cases of OSCC. Increase expression of P63 was found with increasing severity of dysplasia	F

[37]	OSCC, OPMD	349	CD44, CD31, CXCR4, SDF‐1	All markers CD44, CD3 (micro vesicular density), CXCR4, SDF‐1 demonstrated an increasing expression with disease severity in relation to histopathological and dysplasia grading	F

[38]	OLP, NOM	10	E‐cadherin, Vimentin, CK19, b1 integrin, Nestin, STAT1, STAT3	E‐cadherin expression was decreased but vimentin expression was increased in the OLP. Localization of the stem cell markers among the epithelial layers varied between NOM and OLP. Nestin was absent in NOM but tested positive in 80% of OLP samples	F

[39]	OSCC, OLP‐with MT OLP‐without MT NOM	101	ALDH1	Expression of the marker was increased from normal oral mucosa, untransformed OLP, malignant transformed OLP to OSCC	F

[40]	OSCC, OED NOM	385	ALDH1, CD271, CD44, CD24	Except CD271, other markers ALDH1, CD24 and CD44 were increased in OSCC compared to NOM. The intensity of ALDH1 and CD24 markers correlated with increased oral epithelial disease severity	F

[41]	OSCC, leukoplakia	60	CD44, SALL‐4	CD44 expression was associated with the degree of dysplasia in oral leukoplakia. SALL4 expression was negative in all cases of epithelial dysplasia. In OSCCs, SALL 4 expression was associated with degree of differentiation	F

[42]	OSCC, OED, NOM	34	CD44, P63, P53, P73	p63, uniformly expressed throughout the basal and progenitor layers of NOM and in undifferentiated areas of OED and OSCC. Variations in the localization of the markers in different groups is described	G

[43]	HNSCC, OED, NOM	75	FOXM1	Total FOXM1 mRNA expression was low in NOM, a trend of progressive increase with disease severity was observed	F

[44]	NOM, Hyperkeratotic (OHK) and OLP	13	K15, Beta 1 integrin, alpha6 integrin, NG2, Notch 1, MCSP	MCSP and alpa integrin localized in the basal cels at the tips of the papillae indicating the localization of stem cell population. K15 was downregulated in OLP whereas α6, β1 and MCSP were upregulated in both OLP and OHK. NG2 remained unchanged and notch 1 was absent in all samples	F

[45]	OED, NOM	72	Ki‐67, p63, p53, CK19	Ki‐67 labeling index in the basal and supra‐basal layers and that of p63 in the basal layer showed a significant difference between low‐ and high‐grade OED. The variations of the localization of the markers described	F

[46]	OSCC, OED, NOM	40	CD44, SNA‐1	Use of a markers integrated with clinical parameters or SNA‐1 with automated image analysis improved the accuracy to >85%, while multiplexed 2‐marker panel analysis further improved it to >90%	G

[47]	OSCC, OED, OSMF	60	CD44	OSCC showed the highest expression, followed by OED and OSMF with a statistically significant difference	G

[48]	OSCC, OLP, NOM	180	SOX II	Sox11 is upregulated in OLP–associated OSCC–tissues, and associated with poor prognosis in OSCC. DNA methylation regulates Sox11 expression in OLP–associated OSCC, Sox11 drives OSCC cell proliferation through PI3K/AKT signaling activity	G

[49]	OSCC, OED, NOM	70	SOX2	Expression of the marker associated with disease severity of OED	F

[50]	OSCC, OSMF, NOM	135	CD44, CD133	Increased expression of markers associated with disease severity. Multivariate regression analysis demonstrated a significant association between CD44 and CD133 positivity and increased IL‐1β levels	F

[51]	OED, NOM	50	ALDH1, SOX2, CD44, OCT4	ALDH1 and SOX2 demonstrated higher expression in OED with statistical significance. There were no significant association between HPV (p16 expression) and CSC markers	F

[52]	OSCC, OED, NOM	137	Musashi‐1 and CD133	CD133 and Musashi‐1 expression was significantly associated with degree of dysplasia and differentiation of OSCC. A statistically significant positive correlation was found between CD133 and Musashi‐1 in OSCC	F

[53]	OSCC, leukoplakia, OLP, NOM	156	Oct4 and Sox2	Co expression of both markers was evident in OPMD, and epithelial non‐cancer tissues adjacent to the OSCC and primary sites of OSCC but was not evident in NOM	F

[54]	Leukoplakia, OLP, NOM	59	ALDH‐1/2	Significantly higher expression of ALDH1&2 was noticed in the leukoplakia group than in the lichen planus group and in the erosive lichen planus group than in the mildly and nondysplastic leukoplakia group	F

[33]	OSCC, OLP	51	CD147	Increased expression of the marker was observed with increasing disease severity in OED, but not associated with degree of differentiation in OSCC	F

[55]	OSCC, OSMF, NOM	120	OCT 3/4, SOX 2	Significant increased expression of both markers in OSMF and OSCC	F

[56]	OSCC, leukoplakia with and without OED, NOM	55	CD44, TGF‐β	CD44 expression gradually increased from normal mucosa, nondysplastic, dysplastic to OSCC with significant difference. A positive significant correlation between the expression of CD44 and TGF‐B markers	G

[57]	OSCC, OED, NOM	321	FAM3C, PD‐L1, VISTA, B7‐H4, Slug, SOX2 and ALDH1	Expression of FAM3C in OSCC was significantly higher compared to OED and NOM. Expression of FAM3C was positively correlated with PD‐L1, VISTA, B7‐H4, Slug, SOX2 and ALDH1	F

[58]	OSCC, OLP, NOM	110	CD133	Significantly higher expression in progressing lesions than nonprogressing lesions. Absent in NOM, but it positively expressed in the 100% cases of OSCC	G

[59]	OSCC, OPMD, NOM	79	CD44 and ALDH1	Significant overexpression of ALDH1 in high risk OPMD with low risk OPMD was observed. CD44 expression decreased from NOM to OPMDs but increased in OSCC	G

[60]	OLP, NOM	40	CD44	Significantly increased expression in dysplastic OLP, nondysplastic OLP and NOM, respectively	G

[61]	OSCC, OED, NOM	65	SOX2, OCT4, WNT5A	SOX2 demonstrated significantly increased expression with disease severity. WNT5A positivity significantly increased from OED towards OSCC	F

[62]	OSCC associated with OSMF, OSMF, NOM	112	β1 integrin	Localization of the marker among the cell layers varied among groups. Significant difference was observed with the staining index among groups. The non dysplastic epithelium of OSMF with severe atrophy showed expression of the marker	F

[63]	OSCC, OED, NOM	189	ABCG2 and Bmi‐1	An association between disease severity and ABCG2 and Bmi‐1 immuno‐staining intensity was observed. Good correlation of RT‐PCR results for ABCG2 was reported but not Bmi‐1	F

[64]	OSCC, leukoplakia, NOM	203	p75NTR and Ki‐67	Ki‐67 positive cells were increased in association with the severity of OED, similar pattern was not evident in p75NTR. p75NTR was associated with poor survival in OSCC	F

[65]	OSCC, leukoplakia, NOM	203	Np63, Ki‐67, CK14	ΔNp63‐LI was significantly increased with the severity of OED, Ki‐67 and CK14 were also significantly increased with the severity of OED	G

[66]	Leukoplakia, NOM	18	K19, c‐Myc	Expression of both markers were lower in leukoplakia compared to NOM, where K19 decrease was significant	F

[67]	Leukoplakia, OSCC, NOM	57	DAPK‐1	DAPK‐1 expression was statistically significantly higher in leukoplakia without dysplasia/mild dysplasia compared to moderate/severe dysplasia and OSCCs. Higher expression in NOM	G

[68]	Leukoplakia, lichen planus (LP), NOM	59	OCT3‐4 and SOX2	OCT3‐4 was not expressed in any sample. SOX2 expression was statistically significantly higher in erosive type LP than reticular LP.SOX2 expression was statistically significantly higher in mild/nondysplastic leukoplakia than reticular LP	G

[69]	OSMF, NOM	60	CD44	Mean CD44 was lower in OSMF compared to NOM without statistically significant difference. Mean CD44 levels decreased with advancing grades of OSMF	F

[70]	OED, OSCC	40	SOX2	Mean labeling index of SOX2 was higher in OSCC than OED. SOX2 significantly increased from mild to severe OED	F

[71]	Oral lichen planus (OLP), leukoplakia, NOM	59	CD147	Increased expression in the overall lichen planus group versus leukoplakia and NOM. Within OLP subtypes, erosive lichen planus exhibited significantly higher expression than reticular form. CD147 expression was also significantly higher in reticular lichen planus compared with moderately and severely dysplastic leukoplakia	F

[72]	OSCC, OED, oral epithelial hyperplasia (OEH)	58	Snail, E‐Cadherin, CD44, CD133	Nuclear snail expression was detected in all groups, with increased intensity in dysplastic epithelium and highest expression at the invasive front and poorly differentiated regions of OSCC. Loss of E‐cadherin increased stepwise from OEH to OED and OSCC, with significantly lower mean scores in OSCC) compared with OED and OEH. CD44 showed membranous expression across all groups, but mean expression levels did not differ significantly. CD133 expression increased progressively from OEH to OED and OSCC, with significantly higher scores in OSCC compared with premalignant groups	F

[73]	OSCC, premalignant oral lesions, NOM	50	CD44, CD24	CD24 was higher in OSCC group with statistically significant difference in both tissue and blood samples. CD44 was higher in tissue samples but not in blood samples	F

[74]	Leukoplakia, NOM	89	nanog	AG + GG genotypes show an increased frequency in the oral leukoplakia group with a significant difference. Individuals with the AG + GG genotype have a 3.063 higher chance of developing oral leukoplakia compared to individuals with the AA genotype	F

[75]	Leukoplakia (with dysplasia), NOM	84	SOX	No statistically significant association was found between SOX genotypes and IHC expression in leukoplakia	F

[76]	OSCC, actinic cheilitis (AC), oral lichen planus, leukoplakia, NOM	145	Differentiated embryonic chondrocyte‐1 (DEC1), CD44	CD44 expression significantly increased across OPMD and OSCC compared to NOM, DEC1 expression was consistent across lesion types and dysplasia levels. CD44 expression was the highest in AC and OSCC, underscoring its potential role as a progression marker	F

[77]	OSCC, inflammatory gingival hyperplasia, NOM	60	ABCB5	Normal mucosal epithelium exhibited ABCB5 predominantly in the basal layer; inflammatory hyperplasia in the basal, parabasal, and spinous layers; and in OSCC throughout the entire thickness of the epithelium and in invasive islands except for superficial keratin. No significant difference in staining intensity between inflammatory hyperplasia and NOM, but a significantly stronger expression in OSCC was observed compared to other two groups	F

*Note*: QA was conducted using JBI checklists, for analytical cross‐sectional studies 8–7 satisfactory criteria were rated as G, good, 6–4 as F, fair, and 3 and below as P, poor. For case control studies, 10–8 satisfactory criteria were rated as G, good, 7–5 as F, fair, and 4 and below as P, poor.

Abbreviations: HNSCC, head and neck squamous cell carcinoma; MT, malignant transformation; NOM, normal oral mucosa; OED, oral epithelial dysplasia; OLP, oral lichen planus; OSCC, oral squamous cell carcinoma; OSMF, oral submucous fibrosis; QA, quality assessment.

Majority of the studies reported increased expression of the stem cell markers in OPMD compared to normal oral mucosa (NOM). These markers were associated with increasing severity in OED (CD44, ALDH1, HELLS, TARIF, SOX2, NANOG, and CD147); few markers demonstrated a decreasing trend with increasing severity of OED (Nestin, E cadherin, PanCK, DPAK1, and K19). Evidence on the variation of the localization of the stem cell markers within the cellular layers of the epithelium were described in some studies [27, 42, 45, 93].

To assess the potential of the stem cell markers to predict the risk of MT of OPMD, evidence from studies with longitudinal analysis is paramount. Out of the studies included in this review, only a minority (*n* = 11) reported evidence on the association of stem cell biomarkers with follow‐up data. Stem cell markers reported in these studies were, ALDH1, Bmi‐1, OCT4, nEGFR, ABCG2, SOX2, CD133, PIWIL2, CD44, and Podoplanin. Details and findings of these are summarized in Table 2.

**Table 2:** Observational studies reporting stem cell biomarkers in clinical samples with longitudinal analysis.

Study reference	Study groups	Sample size (total)	Stem cell markers	Follow up duration (months)	Study findings	QA
[94]	OSCC, OED	80	ALDH1, Bmi1, and OCT4	10–53 mean 33.3	All the markers increased significantly in OSCC compared to OED. ALDH1and OCT4 were associated with a poor survival in OSCC	P

[95]	OL, OE	50	nEGFR and ABCG2	25–134 median 63	Odds ratio for MT was 8.4 for nEGFR expression, while for ABCG2 expression was 2.2, for both markers was 12.8. Co‐expression of both markers is associated with MT	F

[96]	OED	60	SOX2, podoplanin	6–24	There was significant association of both marker expressions with the degree of dysplasia, the association of their expression with MT did not reach statistical significance	P

[97]	OLP	96	Bmi‐1	10–175 mean 54	The risk of MT in patients with positive expression was significantly higher than those with negative expression with an odds ratio of 20.7	F

[98]	Erythroplakia	34	Bmi‐1 ALDH1	8–68 mean 38	In univariate analysis both markers were significantly associated with MT, in multivariate analysis only ALDH1 was significantly associated with increased risk	F

[39]	OLP	101	ALDH1	12–219 mean 54–60	Increased ALDH1 expression was significantly associated with MT with adjusted odds ratio of 6.7 in multivariate analysis	F

[99]	Leukoplakia	79	ALDH1 podoplanin	Mean 40	Multivariate analysis revealed that ALDH1 and podoplanin was associated with 3.02‐ and 2.62‐fold increased risk of MT. The risk was considerably higher in cases with expression of both markers	F

[100]	Leukoplakia	141	CD133, ALDH1	Mean 65	48.1% patients with ALDH1‐positivity developed OSCC compared with 12.6% those with ALDH1‐negativity Multivariate analysis revealed that ALDH1 and CD133 expression was associated with 4.17‐fold and 2.86‐fold increased risk of MT	F

[101]	Leukoplakia	135	ABCG2, Bmi‐1, 2	12–240 mean 65	ABCG2 and Bmi‐1 expression was associated with a 3.24‐fold and 4.03‐fold increased the risk of MT	F

[102, 103]	Leukoplakia	101	PIWIL2	NR	Univariate and multivariate analysis revealed grade of dysplasia and PIWIL2 were significant predictors of MT	P

[104]	OED, OSCC	37	CD24, CD44	9–193 months	No statistically significant difference was found with sex, age, grade, stage, recurrence, regional recurrence, metastasis, overall survival and disease‐free survival	F

*Note*: QA was conducted using JBI checklist for cohort study designs, 11–9 satisfactory criteria were rated as G, good, 8–6 as F, fair, and 5 and below as P, poor.

Abbreviations: MT, malignant transformation; NR, not reported; OE, oral erythroplakia; OED, oral epithelial dysplasia; OL, oral leukoplakia; OLP, oral lichen planus; OSCC, oral squamous cell carcinoma; QA, quality assessment.

When summarizing the evidence from longitudinal data, four studies reported that ALDH1 was significantly associated with MT of three clinical subtypes of OPMD, these were erythroplakia [98], OLP [39], and leukoplakia [99, 100]. Co‐expression of two markers reported significant association with MT, these were nEGFR and ABCG2 [95], and ALDH1 with podoplanin [99]. Regarding Bmi‐1, there was conflicting evidence. Two studies did not find Bmi‐1 significantly associated with MT [94, 98] for OED and erythroplakia, while two other studies reported significant association of Bmi‐1 with the MT of OLP [97] and leukoplakia [101]. Main observation was the significant lack of prospectives designs, except one [96]; rest of the studies employed a retrospective analysis from archived tissue samples.

### 3.3. Evidence From Cell Culture Experiments

A total of 17. 5% (*n* = 17/97) of the included studies employed cell culture experiments. Out of these, four studies provide evidence on the effectiveness of stem cell therapies to prevent the disease progression of OPMD. Few studies used adipose stem cells and their derivatives (exosomes), as a treatment method for oral submucous fibrosis (OSMF), their results demonstrate that adipose stem cells were successful in haltering the progression of this disease at the cellular level [78–80]. Another study explored the use of dental pulp stem cells (DPSC) as a therapeutic modality for OSMF, this study revealed that DPSC demonstrated significant antifibrotic effects and was able to suppress OSMF fibroblasts activity [91]. One study explored the utility of an herbal extract (total glucosides of peony) as a therapeutic agent to attenuate the inflammatory properties of mesenchymal stem cells in OLP [85]. Rest of the studies have reported different stem cell markers, and their molecular and cellular mechanisms that contribute to MT in OPMD. These mechanisms include resistance to apoptosis by extending G2 phase of cell cycle, increasing the proliferative ability, promote destruction of basement membrane, suppression of T lymphocytes, promote angiogenesis, increase stemness properties in normal keratinocytes, and activating downstream signaling molecules such as HELLS, HOXC9, and PI3K‐AkT cellular pathways. Summarized evidence from the studies are provided in Table 3.

**Table 3:** Studies using cell culture experiments.

Study reference	Cell culture experiment	Main findings	QA
[78]	Primary culture of human adipose derived stem cells (ADSC), extraction of exosomes and assessed their therapeutic application in the management of OSMF	ADSC‐Exo was effective in promoting the activities of myofibroblasts, reversing the collagen deposition and myofibroblast trans‐differentiation regulate the TGF‐b pathway to alleviate arecoline‐induced OSMF	F

[79]	Therapeutic potential of adipose derived stem cell exosomes (ADSC‐Exo) in the management of OSMF	Findings from the present study suggest that ADSC‑Exo’s may represent a promising strategy for OSMF treatment by targeting the p38 MAPK signaling pathway	F

[80]	Therapeutic potential of adipose derived stem cell exosomes (ADSC‐Exo) in the management of OSMF	ADSC‐Exo inhibited fibrosis and alleviated OSMF by targeting FOXF1	G

[81]	Analysis of CD44 positive cells in cancer cell lines	CD44 high cells with stem‐like properties exhibit resistance to apoptosis due to extended G2 phase of the cell cycle, suggesting their role in tumor recurrence and potential therapeutic targeting	F

[82]	Normal tissue (OKF6‐TERT2), mild dysplasia (DOK), severe dysplasia (POE‐9n), and OSCC (PE/CA PJ15)	Loss of spatial regulation of CD44 and p75NTR markers correlates with malignancy, suggesting their roles in disease progression	F

[83]	Dysplastic epithelial cell lines (DysMSCTR6, DysMSCTR14, and DysMSCTR16) and fibroblast cell lines (FibroMSCTR12 and FibroMSCTR16)	Increased expression of CD44, CD133, ALDH1A1, and NOTCH1 correlated with dysplasia progression. NOTCH1 inhibition reduced CSC markers and colony formation. Fibroblast niche promoted CSC enrichment and increased proliferation. NOTCH1 plays a critical role in CSC maintenance, but fibroblast‐induced proliferation is NOTCH1‐independent	F

[84]	Premalignant oral lesion‐derived cell lines (DOK), vimentin‐expressing clones	High vimentin‐low E‐cadherin expression significantly correlated with high‐grade dysplasia and lymph node metastasis. High vimentin‐low E‐cadherin expression in early lesions may indicate a high risk of malignant transformation. Vimentin alone is not sufficient for transformation but enhances susceptibility when exposed to carcinogenic stimuli	F

[85]	Assessing the effect of an herbal extract total glucosides of peony—TGP on the mesenchymal stem cells (MSC) derived from OLP	MSCs from disease express more inflammatory cytokines than MSCs from normal controls. TGP has a significant effect on decreasing pro‐inflammatory cytokines and increasing anti‐inflammatory mediators via the miR‐124‐3p/STAT3 pathway. We suggest that TGP may be a new and safe drug for improving the function of MSCs	G

[86]	Mesenchymal stem cells (MSC) from leukoplakia and normal controls	Expression of Col IV was decreased and MMP‐9 was increased by MSC. The imbalance between regenerative and metabolic self‐regulatory functions of MSCs from oral leukoplakia may be related to the disease progression	G

[87]	Mesenchymal stem cells (MSC) derived from oral lichen planus and normal controls	MSCs from OLP may participate in immunomodulation by suppressing T lymphocytes proliferation via (indoleamine 2,3‐dioxygenase‐ IDO) activity	G

[88]	MSC derived from OSCC, oral leukoplakia and controls	The present study revealed that exosomes derived from the MSC of oral leukoplakia and OSCC enhanced angiogenic activity in vivo and in vitro	F

[89]	Head and Neck cell lines‐ HN12/HN13, nontumor keratinocytes	SK2 overexpression increases the stem cell properties in normal keratinocytes and thereby can contribute to the OSCC development	G

[90]	Primary culture of MSC derived from OSCC, oral leukoplakia (OLK) and normal oral mucosa; HOK, DOK, leuk1, Cal27 cell lines	Differentially expressed genes of OSCC–MSC compared to OLK–MSC were primarily associated with the PI3K‐Akt signaling pathway and tumor‐related pathways. OSCC–MSC exhibited stronger migratory and invasive abilities compared to Cal27. MSC from OLK, OSCC require higher concentrations of photodynamic therapy treatment than MSC of the same tissue origin	F

[38]	Primary culture of normal epithelial cells, exposed to IFN‐γ and the expression of stem cell markers were compared before and after	The results showed that b1 integrin, a6 integrin, and nestin were elevated under culture condition with IFN‐γ exposure	F

[43]	Premalignant (SVpgC2a) and malignant (SqCC /Y1, SCC25) cell lines were exposed to nicotine and arecoline and FOXM1 expression and its functional pathway was analyzed	Nicotine showed dose dependent activation of FOXM1, but this activity was absent for arecoline. CEP55 and HELLS were downstream targets of FOXM1	F

[91]	Primary culture of fibroblasts from OSMF patients, dental pulp stem cells (DPSC) from healthy donors	DPSCs exhibit strong antifibrotic properties by suppressing fibroblast proliferation, inhibiting collagen contraction, and reducing TGF secretion	F

[92]	A cell culture experiment was conducted using DOK, HSC3 cell lines to study the role of HOXC9 in the MT of leukoplakia	HOXC9 induces the acquisition of cancer stem cells (CSCs) and epithelial‐to‐mesenchymal transition	F

*Note*: QA was conducted using JBI checklist for analytical cross section study designs, 8–7 satisfactory criteria were rated as G, good, 6–4 as F, fair, and 3 and below as P, poor.

Abbreviations: NOM, normal oral mucosa; OLP, oral lichen planus; OSMF, oral submucous fibrosis; QA, quality assessment.

### 3.4. Evidence From Animal Model Experiments

There was a total of 10.3% (*n* = 10/97) experimental studies using animal models. Out of these, two studies used DMBA, five studies used 4NQO to induce the OPMD disease models and three studies used local application of arecoline to induce OSMF disease model. Bruna et al. [105] provide evidence that mesenchymal stem cells can be used to halter the disease progression in to OSCC in a time and dose dependent manner [105], going a step further to their experiment, another study employed exosomes derived from mesenchymal stem cells and transfected them with miR‐185, and explored their ability to halter the disease progression in OPMD [102]. Another study used adipose derived stem cells in a similar experiment as a therapeutic measure for OSMF [106]. Complimentary to the evidence from cell culture experiments, treatment with DPSCs could mitigate the action of OSMF fibroblasts, two studies demonstrated its potential to halter disease progression of OSMF in animal models [91, 107, 108]. Findings of the studies using animal model experiments are presented in Table 4.

**Table 4:** Experimental studies using animal models.

Study reference	Animals	Disease model	Main findings	QA
[102]	6–8 weeks old male hamsters (*n* = 60)	DMBA induced OPMD	Genetically modified (transfected with MiR‐185) extracellular vesicles derived from mesenchymal stem cells delay the progression of OPMD	F

[23]	5‐weeks old female C57BL/6 mice (*n* = 80)	4NQO induced oral leukoplakia	Preliminary evidence on PD‐1 blockade (via anti‐PD‐1 antibody) can active CD8^+^ T cells to delay the disease progression	F

[109]	Male wistar rats (*n* = 27)	4NQO induced OED and OSCC	p75 neurotrophin receptor (p75NTR) is a cancer stem cell biomarker appearing in the early stage of oral carcinogenesis	F

[110]	5‐week‐old female C57BL/6 mice (*n* = 15)	4NQO induced OED	Human dental pulp stem cells activate mTOR signaling pathway through mitochondrial transfer, and promote the MT of OED	F

[105]	8 weeks old male hamsters (*n* = 180)	DMBA induced OSCC	Systemic administration of MSC does not accelerate lesion progression. MSCs may suppress OSCC tumor growth depending on dose and lesion stage	F

[111]	6 weeks old female Sprague‐Dawley Rats (*n* = 18)	4NQO induced OED and OSCC	The proportion of MSC increased with disease severity, without alteration in stemness properties. MSCs showed increased immunosuppression capacity on T cell proliferation. MSCs was positively correlated with Ki67 expression	G

[89]	12 weeks old male BALB/c‐Mice (*n* = 20)	4NQO and cells (NOK‐SI HN12) induced OSCC	Sphingosine kinase 2 (SK2) is a biomarker indicating the stemness, have multiple roles in tumor initiation and progression through various cellular pathways. SK2 overexpression increases the stem cell properties in normal keratinocytes and thereby can contribute to the OSCC development	G

[107]	Male Swiss albino mice (*n* = 40)	Arecoline induced OSMF	Human dental pulp MSC secretome was used as a treatment for the disease model compared to control group. This method demonstrated significant antifibrotic, antioxidant, and regenerative effects and was able to reverse ANE–induced OSMF in mice	F

[106]	6–7 weeks old C57BL/6 mice (*n* = 90)	Arecoline induced OSMF	Adipose derived stem cells (ADSC) exosomes were used as therapeutic agent for OSMF mouse model. It significantly mitigated fibrosis level, reducing alpha‐SMA and Collagen I expression, and downregulated tissue TGF‐beta and autophagy markers. ADSCs‐exosomes attenuates myofibroblast transformation by inhibiting autophagy via the TGF‐beta/Smad2 axis	G

[108]	8‐week‐old male Sprague‐Dawley rats (*n* = 30)	Arecoline induced OSMF	Dental pulp stem cells (DPSC) used as a therapeutic agent for OSMF compared to corticosteroids in a rat model. DPSCs demonstrated superior therapeutic effects compared with the positive control (glucocorticoids), including reducing collagen deposition and promoting blood vessel regeneration. DPSCs mediated immune homeostasis primarily by regulating the numbers of KRT19 + MIF + epithelial cells and via epithelial‐stromal crosstalk	G

*Note*: QA was conducted using JBI checklist for analytical cross sectional study designs, 8–7 satisfactory criteria were rated as G, good, 6–4 as F, fair, and 3 and below as P, poor.

Abbreviations: MSC, mesenchymal stem cell; QA, quality assessment.

Interestingly, one study employed a randomized controlled trial design [112], and this study used stem cell marker (CD133) as a surrogate end point to assess the effectiveness of two treatment methods for the management of OLP. Results showed that both treatment methods (topical pimecrolimus and betamethasone application) were able to reduce the CD133 stem cell population in OLP.

## 4. Discussion

The conventional understanding of stem cells as components of embryonic development is being replaced with the expanding knowledge on their emerging roles in diseases, including cancer. Research evidence on the involvement of stem cells in therapy resistance, immune evasion, and tumor microenvironment are mounting [113]. Further, manipulation of stem cells and their derivatives (transfection of exosomes or extracellular vesicles derived from stem cells with micro‐RNA and therapeutic agents) and their use in precision medicine and targeted therapy is gaining attention [114]. In this backdrop, the current systematic review was conducted to identify and appraise contemporary evidence on the role of stem cells and stem cell biomarkers in OPMD and their MT. Results of the current systematic review revealed that stem cells are employed for several main applications in relation to OPMD. These were as potential biomarkers for risk assessment and prediction of MT in OPMD, as markers of disease severity of OED, as therapeutic agents and surrogate end points to assess the efficacy of treatment methods.

Dysplasia grading is subjected to limitations by inter and intra examiner variation; hence, the histopathological grading of dysplasia alone is insufficient to predict the MT of OPMD [3, 115]. Observational studies included in this review have explored the utility of stem cell markers as biomarkers of disease severity in OED. Immunohistochemistry appeared to be the common technique used for identification of stem cell markers in tissue samples. However, an array of heterogenous stem cell markers (e.g., HELLS, CD44, CD24, TRAF1, Bmi‐1, ALDH1, Lin28B, NANOG, NICD, Hes‐1, c‐Myc, PD‐1, PD‐L1, TIM‐3, CTLA‐4, CD8, and CD147) have been reported. Even though these markers can be used to demonstrate the progressive increase of the subpopulation of CSCs with increasing severity of OED and their variability in localization within the cellular layers, there remains ambiguity for a single stem cell biomarker as a prognostic marker for OED with substantial evidence. In contrast, algorithms incorporating multiple data inputs such as markers of stem cell population, clinical features, and image analysis has demonstrated a successful approach for risk assessment and prognosis of OED [46].

Current evidence from longitudinal studies suggests that ALDH1 as a promising biomarker that could predict the MT of several clinical subtypes in OPMD. Further research is needed to understand the true potential of Bmi‐1 as a risk assessment biomarker for OPMD, as conflicting results were reported in different studies. However, most studies providing longitudinal data were conducted as retrospective analysis from archived tissue samples. Our results indicate a dire need for studies with prospective designs on the use of stem cell markers as risk assessment tools for early detection of MT in different clinical subtypes of OPMD.

Evidence from both cell culture and animal experiments have uncovered several downstream pathways triggered by stem cell populations that facilitate the development of hallmarks of carcinogenesis in OPMD. These can be used as potential targets to develop therapeutic agents to halt disease progression. Antiparallel to the stem cell function in immune modulation to promote carcinogenesis, blockade of immune checkpoint inhibitor PD‐L1 was able to activate T cells and promote immune destruction [23].

Abnormal fibrosis with alteration of structure and cross linking of collagen is hallmark in the pathogenesis of oral submucous fibrosis (OSMF). Adipose derived stem cells and their exosomes were effective in haltering the fibrosis through different cellular mechanisms in OSMF [78–80]. Furthermore, DPSCs were able to reverse the fibrotic changes in OSMF in both cell culture and animal experiments [91, 107, 108]. These effects may be due to the inherent regenerative capacity of stem cells and their ability to promote growth and generate new cells and tissues in their vicinity. Stem cell derived exosomes are gaining popularity as vehicles to transport micro‐RNA and pharmacological agents to disease cells. Compared to their application as risk assessment biomarkers for OPMD, preliminary but promising evidence suggests a more critical role of stem cells and their derivatives in precision medicine for OPMD. Future studies must focus on assessing the efficacy of this method in prospective clinical trials compared to conventional therapies for OPMD. However, clinical translation will be challenging due to high cost and technique sensitivity.

There is substantial evidence that CSCs increase progressively with the increase severity of OED, with complimentary studies providing evidence on the downstream molecular pathways related to CSCs and their involvement to promote hallmarks of carcinogenesis. Therefore, it is justified to use stem cells as a marker to assess the efficacy of different treatment modalities on OPMD. Ezzatt and Helmy [112] in their randomized controlled trail used stem cell marker as a surrogate end point to assess the efficacy of two treatment methods [112]. In addition to effectiveness in reducing the clinical symptoms, the theraputic effect at the cellular level was demonstrated by a reduction of CSC population.

Common limitations of the included studies were the absence of a matched control group for comparison, inadequate sample size in different OED grades, and lack of analysis on the associations between sociodemographic and risk factors with stem cell marker expression. In studies using retrospective analysis, study groups were selected based on outcome (e.g., malignant transformed and nontransformed groups). Therefore, groups were not similar in terms of age, gender, and degree of dysplasia which could have influenced study findings as co‐founding variables. To provide accurate understanding on the application of stem cell markers as risk assessment tools for early detection MT in OPMD, studies with matching groups without the outcome (MT), and prospective follow up are recommended. In addition, combination of several data sources such as CSC markers, clinical features, and histopathological images with advance technologies such as machine learning may provide a more holistic and robust strategy for the risk assessment of OED. Downstream molecular pathways facilitated by CSCs may be explored as potential targets to develop pharmacological agents to mitigate disease progression in OPMD.

### 4.1. Critical View

Observational studies, cell culture, and animal experiments provide significant evidence on the applications of stem cells in OPMD. Particularly, increased expression of stem cell markers was related to increasing severity in OED. Together with increased expression, variations in tissue level localization and molecular mechanisms of stem cells that contribute to oral carcinogenesis have been reported. Current evidence indicates that ALDH‐1 and Bmi‐1 as promising stem cell biomarkers for risk assessment of OPMD with further validation. Research evidence, especially from prospective cohort studies, are needed to establish stem cell markers as risk assessment tools for early detection of MT in OPMD. Most promising application is the use of stem cells and their derivatives in precision medicine and as therapeutic agents to halt the progression of OPMD. However, there are only limited number of studies and preliminary level evidence on this application. This review could not retrieve any clinical trials on the application of stem cells as therapeutic modality for OPMD. Stem cell markers can be used as surrogate end points in clinical trials comparing different treatment modalities for OPMD.

## Author Contributions

**Nadisha S. Piyarathne**: conceptualization, data curation, validation, analysis, writing – the original draft. **Gayani S. Nawarathna and W. J. Wijesingha**: literature search, data curation, validation. **Udari Abeyasinghe**: protocol design. **P. V. Kalani Hettiarachchi**: data curation, validation, review the manuscript.

## Acknowledgments

Authors are grateful to all original authors of the studies included in this systematic review. For the purpose of open access, the author has applied a Creative Commons Attribution (CC BY) license to any author accepted manuscript arising from this submission. The authors declare that they have not used any AI–generated content during the preparation of this manuscript

## Funding

This study did not receive any external funding.

## Ethics Statement

The authors have nothing to report.

## Consent

The authors have nothing to report.

## Conflicts of Interest

The authors declare no conflicts of interest.

## Data Availability

All data generated from this study will be available upon reasonable request from the corresponding author.
